# Acceptability and Potential Effectiveness of eHealth Tools for Training Primary Health Workers From Nigeria at Scale: Mixed Methods, Uncontrolled Before-and-After Study

**DOI:** 10.2196/24182

**Published:** 2021-09-16

**Authors:** Joseph Paul Hicks, Matthew John Allsop, Godwin O Akaba, Ramsey M Yalma, Osasuyi Dirisu, Babasola Okusanya, Jamilu Tukur, Kehinde Okunade, David Akeju, Adegbenga Ajepe, Okey Okuzu, Tolib Mirzoev, Bassey Ebenso

**Affiliations:** 1 Nuffield Centre for International Health and Development University of Leeds Leeds United Kingdom; 2 Academic Unit of Palliative Care Leeds Institute of Health Sciences University of Leeds Leeds United Kingdom; 3 Department of Obstetrics and Gynaecology University of Abuja Gwagwalada Abuja Nigeria; 4 Department of Community Medicine University of Abuja Gwagwalada Abuja Nigeria; 5 Population Council Abuja Nigeria; 6 Department of Obstetrics and Gynaecology College of Medicine University of Lagos Lagos Nigeria; 7 Aminu Kano Teaching Hospital Kano Nigeria; 8 Department of Sociology College of Medicine University of Lagos Lagos Nigeria; 9 Instrat Global Health Solutions Abuja Nigeria; 10 Department of Global Health and Development London School of Hygiene & Tropical Medicine London United Kingdom

**Keywords:** primary health worker training, digital health technology, eHealth, video-based training, maternal and child health, Nigeria, mobile phone

## Abstract

**Background:**

The in-service training of frontline health workers (FHWs) in primary health care facilities plays an important role in improving the standard of health care delivery. However, it is often expensive and requires FHWs to leave their posts in rural areas to attend courses in urban centers. This study reports the implementation of a digital health tool for providing video training (VTR) on maternal, newborn, and child health (MNCH) care to provide in-service training at scale without interrupting health services. The VTR intervention was supported by satellite communications technology and existing 3G mobile networks.

**Objective:**

This study aims to determine the feasibility and acceptability of these digital health tools and their potential effectiveness in improving clinical knowledge, attitudes, and practices related to MNCH care.

**Methods:**

A mixed methods design, including an uncontrolled pre- and postquantitative evaluation, was adopted. From October 2017 to May 2018, a VTR mobile intervention was delivered to FHWs in 3 states of Nigeria. We examined changes in workers’ knowledge and confidence in delivering MNCH services through a pre- and posttest survey. Stakeholders’ experiences with the intervention were explored through semistructured interviews that drew on the technology acceptance model to frame contextual factors that shaped the intervention’s acceptability and usability in the work environment.

**Results:**

In total, 328 FHWs completed both pre- and posttests. FHWs achieved a mean pretest score of 51% (95% CI 48%-54%) and mean posttest score of 69% (95% CI 66%-72%), reflecting, after adjusting for key covariates, a mean increase between the pre- and posttest of 17 percentage points (95% CI 15-19; *P*<.001). Variation was identified in pre- and posttest scores by the sex and location of participants alongside topic-specific areas where scores were lowest. Stakeholder interviews suggested a wide acceptance of VTR Mobile (delivered via digital technology) as an important tool for enhancing the quality of training, reinforcing knowledge, and improving health outcomes.

**Conclusions:**

This study found that VTR supported through a digital technology approach is a feasible and acceptable approach for supporting improvements in clinical knowledge, attitudes, and reported practices in MNCH. The determinants of technology acceptance included ease of use, perceived usefulness, access to technology and training contents, and the cost-effectiveness of VTR, whereas barriers to the adoption of VTR were poor electricity supply, poor internet connection, and FHWs’ workload. The evaluation also identified the mechanisms of the impact of delivering VTR Mobile at scale on the micro (individual), meso (organizational), and macro (policy) levels of the health system. Future research is required to explore the translation of this digital health approach for the VTR of FHWs and its impact across low-resource settings to ameliorate the financial and time costs of training and support high-quality MNCH care delivery.

**Trial Registration:**

ISRCTN Registry 32105372; https://www.isrctn.com/ISRCTN32105372

## Introduction

### Background

After more than a decade of rapid development, approaches using digital health technologies are gaining prominence as a means of addressing health system challenges for improving access to and the quality of health service delivery. However, a need to continue the development of evidence bases still exists [1]. Digital technologies can be used to strengthen health systems and work toward achieving universal health coverage [2]. The onus is now on governments to recognize the importance of digital technologies used in this way, with the World Health Organization’s (WHO) member states endorsing this approach at the 71st World Health Assembly [3].

The WHO defines digital health technologies as a salient field of practice for using routine and innovative forms of information and communications technology (ICT) to address health needs [4]. This has been recognized through an ICT-related target in Millennium Development Goal 8, in the proceedings of the World Health Assembly resolution on eHealth (WHA58.28), alongside a recent World Health Assembly resolution passed on Digital Health (A71-R7) urging the member states “to prioritize, as appropriate, the development, evaluation, implementation, scale-up and greater use of digital technologies, as a means of promoting equitable, affordable and universal access to health for all” [5]. Regionally, in Sub-Saharan Africa, there have been diverse implementations of digital health tools, including those targeting improvements in the use of health care services via reminder text messages, teleconferencing, data management, and information dissemination [6]. A large proportion of published literature on the use of digital health tools specific to maternal, newborn, and child health (MNCH) services in low- and middle-income countries (LMICs) is based in Sub-Saharan Africa [7,8]. In Nigeria, there have been numerous government-led initiatives exploring the role of digital technologies in improving MNCH services, which continue to be the most explored aspects. A recent review of the landscape and inventory of ICTs for health in Nigeria revealed that more than 100 different ICT projects were implemented across the country to strengthen a range of health system functions [9]. The focus on the different program areas included 63 ICT projects on MNCH (eg, providing information to women on healthy living and clinical decision support to their caregivers), 36 projects on health system functions (eg, improving health information systems), 13 projects on health worker training and education, and 6 projects on health financing. This proliferation of ICT programs commenced in 2013 when the Federal Government of Nigeria prioritized ICTs as a strategy for achieving the targets of a *Saving One Million Lives* initiative that aimed at broadening universal access to essential primary health care (PHC) services for vulnerable mothers and their infants [10]. This government-led digital health coordination mechanism under the *Saving One Million Lives* initiative is an example of a national-level institutionalization of digital health [11].

In 2019, the WHO released guidelines on digital health interventions for health system strengthening [4]. Following the critical evaluation of the evidence on emerging digital health interventions for health system improvements, they made multiple recommendations for interventions. These included providing training and educational content digitally, with evidence suggesting that digital apps may increase both health workers’ knowledge, the acceptability of the training and educational content to workers, and the feasibility of delivering it. Such an approach can help to address health system constraints, including training deficits and rural reach, which are known to have a detrimental impact on MNCH services. Training delivered in this way holds promise for transforming education and training for health providers and patients by virtue of its potential to (1) reach users across large geographical areas, (2) increase the speed of delivery of training content, and (3) provide learners with the flexibility to study at their own pace and convenience while adapting their learning to their needs and preferences [12]. Even with promising research, there is currently a lack of evidence on the effects of interventions that seek to leverage digital approaches to deliver training and educational content in LMIC contexts, including their impacts on outcomes such as health workers’ performance, skills, and attitudes [4]. This situation is further reflected at the national level in Nigeria, where there is a lack of empirical evaluations on the impact of digital health approaches for health tools in general [9], and in training frontline health workers (FHWs) in remote areas that lack regular telecommunications network connectivity.

### Objectives

This study addresses this gap in the evidence base by outlining the evaluation of a digital health tool to extend tablet-based educational training for FHWs in rural areas of Nigeria via satellite telecommunications technology. The development of digital health tools has been reported elsewhere [13]. To the best of our knowledge, this is the first report on providing an e-learning intervention that uses satellite telecommunication to reach rural FHWs at scale in an LMIC context, with content specifically tailored to the education and training of FHWs on MNCH care. This paper defines a successful scale in digital technology as the institutionalization or embedding of a digital health product into each level of the health system rather than regarding it as a separate activity [11,14]. In this sense, the integration of e-learning into policy, practices, workflows, and daily lives of health workers in multiple states of Nigeria represents the successful scaling of digital technology. In this paper, we aim to report on the feasibility and acceptability of these tools to rural FHWs and their potential effectiveness in improving FHWs’ clinical knowledge, attitudes, and reported practices related to MNCH care.

## Methods

### Study Design

The e-learning study reported here was embedded within a larger project that combined the video training (VTR) and digitization of health data interventions [13]. Only VTR interventions are reported here. The e-learning component of the larger project involved supplying a computer tablet–based VTR app to 126 rural PHC facilities across three Nigerian states: Federal Capital Territory (FCT), Kano state, and Ondo state [13]. The system enabled the transmission of prerecorded, high-quality training videos and other learning content from a remote server to facilitate the training of rural FHWs on MNCH care, further reducing the need for FHWs to travel to metropolitan cities for training. This larger project included a nonrandomized cluster trial examining the impacts of providing eHealth tools and facilitating infrastructure, specifically satellite communication (SatCom) equipment, to enable remote rural PHC facilities for accessing the internet. This was compared with not providing any eHealth intervention, facilitating infrastructure or any internet access, on routine health service data quality and service provision and use. The data reported in this study relate only to intervention sites that had internet access either via existing 3G mobile networks or through SatCom in facilities without 3G connectivity.

For the quantitative component of this study, we used an uncontrolled before-and-after (or pre-post) design to compare whether rural FHWs’ knowledge, attitudes, and reported practices across a range of key MNCH topics changed before and after receiving access to the VTR intervention tools and associated training content. For the qualitative component of the study, we used face-to-face, semistructured in-depth interviews (IDIs) with purposefully selected stakeholders, including FHWs, heads of health facilities, and policy makers, to understand the acceptability, feasibility, and use of computer-enabled VTR to improve health care provision in participating states of Nigeria. IDIs were conducted from February 19 to March 9, 2018 (ie, 12-14 weeks into the implementation of VTR).

### Setting and Participants

The study was conducted across three states in Nigeria (Kano, Ondo, and FCT), as outlined in Table 1. Within each state, health facilities were selected purposively (for the wider project) across local government areas, which were assigned as intervention local government areas. We included a total of 126 health facilities in this study, which were subcategorized according to the National PHC Development Agency criteria as PHC facilities, comprehensive health centers, health posts, and basic health centers, and were unequally distributed in number and type across the three study areas (Table 1).

**Table 1:** Distribution of participating health facilities by their locations in Nigeria (N=126).

Participating states	Region	Population size (2006 census figures) [15]	Participating local government areas	Facility type distribution by state				
				Primary health center (n=91), n (%)	Comprehensive health center (n=6), n (%)	Health post (n=22), n (%)	Basic health center (n=7), n (%)	Total (N=126), n (%)
Ondo state	Western Nigeria	3,460,877 (18th of 37)	Akoko South, Idanre, and Odigbo	58 (63.7)	4 (66.7)	0 (0)	0 (0)	62 (49.2)
Kano state	Northern Nigeria	9,401,288 (1st of 37)	Dawakin Tofa and Sumaila	7 (7.7)	0 (0)	21 (95.5)	7 (100)	35 (27.8)
FCT^a^	Federal territory containing Abuja, in Central Nigeria	1,406,239 (37th of 37)	Gwagwalada and Kuje	26 (28.6)	2 (33.3)	1 (4.5)	0 (0)	29 (23)

^a^FCT: Federal Capital Territory.

PHC facilities, health posts, and basic health centers all provide primary-level care, whereas comprehensive health centers provide secondary-level care. Primary-level facilities often serve as the first point of contact for patients and are mainly staffed by community health extension workers (CHEWs) but have no medical doctors or midwives, whereas secondary-level facilities serve as referral centers and are staffed by CHEWs, medical doctors, nurses, and midwives. In Nigeria, CHEWs are FHWs trained for 2 to 3 years in the schools of health technology to provide basic public health services at primary-level facilities and mainly to assist nurses and midwives in their duties [16]. Our study involved two types of FHWs who were targeted by the intervention: CHEWs, who are present in all four types of health facilities in the study, plus nurses and midwives, who are only present in PHC facilities and comprehensive health centers. In basic health centers and health posts, there was typically just one FHW available for the study (often the facility manager), whereas there were typically at least two FHWs available in PHC facilities and comprehensive health centers, as they usually have a mix of cadres present. Members of the research team recruited all selected FHWs after obtaining permission from their facility managers, explaining the study’s objectives to the FHWs and obtaining their consent to participate. This was followed by an orientation on how to use eHealth interventions.

### eHealth Intervention

The intervention involved providing all recruited facilities with a tablet computer containing a VTR app (VTR Mobile). VTR Mobile allows users to access video, audio, and text-based learning materials through the internet. The educational videos used for this study were developed by Medical Aid Films [17] and Global Health Media Project [18] and accessed via the ORB platform [19] developed by the mPowering FHWs Partnership [20]. The ORB platform hosts high-quality medical content that can be used under a Creative Commons License to train frontline workers via the internet or via downloads to mobile devices. The educational videos provided clear educational content and engaged clinical scenarios focused on MNCH care, specifically antenatal care, basic obstetric care, perinatal care, and postnatal care. We selected the content of the videos in consultation with the relevant state ministries of health. The videos were delivered to the users via a structured VTR mobile program. User log-ins were created and provided by the staff of the eHealth intervention provider, InStrat Global Health Solutions [21], to the study participants to enable them to log in and work their way through the program, which also tracked their progress.

### Quantitative Data Collection and Outcomes

We collected all data via the tablet computers used by the FHWs, with the data automatically uploaded onto remote servers before being accessed by the research team. To assess whether there were any changes in FHWs’ knowledge, attitudes, and reported practices related to MNCH care, following access to the eHealth intervention tools and information, all FHWs accessing the VTR mobile system first took a multiple-choice (48 questions) preintervention test (*pretest*) that assessed their reported MNCH knowledge, attitudes, and reported practices on the following 9 topics: (1) focused antenatal care (5 questions), (2) respectful maternity care (2 questions), (3) warning signs in pregnancy (6 questions), (4) how to use a partograph (5 questions), (5) the prevention of postpartum hemorrhage (5 questions), (6) the management of postpartum hemorrhage in a low-resource setting (8 questions), (7) the manual removal of placenta (5 questions), (8) neonatal resuscitation (5 questions), and (9) how to care for a newborn (7 questions).

The postintervention test (*posttest*) questions were the same as the pretest questions. Questions were aligned to the content included in the e-learning program and the curriculum of the included educational videos. The questions were developed by the research team, which included specialists in practice and training in obstetrics and gynecology in Nigeria. Furthermore, consultation with state governments and policy makers occurred during the study planning to ensure that the curriculum of the e-learning program as well as the pre- and posttest questions aligned with the federal government and WHO guidelines for maternal and child health (eg, staff attitudes and provision of respectful maternity care) and that the pre- and posttest questions were clear and easy to understand. Participants who completed the nine VTR modules were automatically prompted via the tablet to take the posttest. Those who had not completed the posttest after 4 weeks of registering for and starting the intervention received fortnightly mobile telephone reminders from the intervention support staff of InStrat to complete the posttest. Multimedia Appendix 1 outlines the questions asked for each topic. Those who had not completed the posttest by the 18th week received weekly text messages from the intervention support staff. No other tests were conducted outside the pre- and postintervention tests in this study. For each user, the system collected data on whether each question was correctly answered. We then calculated our primary outcome as the overall percentage of questions correctly answered in the pre- and posttests. We also created several secondary outcomes based on the percentage of questions correctly answered in both the pre- and posttests, but within each test topic separately. In addition to the pre- and posttest outcome data, we also collected data on FHWs’ gender, staff type (CHEW or nurses and midwives), facility type (PHC, comprehensive health centers, health post, or basic health centers), SatCom availability at their facility, facility location (Ondo, Kano, or FCT), and the date of their pre- and posttests, which we used to create a variable measuring the number of days between FHWs’ pre- and posttests.

### Statistical Analyses

We calculated that a sample size of 324 would provide >80% power to detect an overall increase of 20 percentage points between the pre- and posttest scores, assuming the most conservative overall prescore of 50%, using a two-sided hypothesis test with a significance level of 0.05 and assuming a modest, typical design effect of 1.5, in the absence of any comparable or pilot data, and 10% loss to follow-up. To describe the characteristics of FHWs and health facilities in our study sample, we produced relevant descriptive statistics. To estimate the change in the overall FHW test score results between the pre- and posttests, we first fitted a multilevel linear regression model with the outcome of test score (including both pre- and posttest scores for every FHW with complete data) and fixed effects for the test period (pre- or posttest), gender (male or female), staff type (CHEW or nurses and midwives [merged due to low sample sizes]), facility type (PHC or comprehensive health centers, or health post and basic health centers [merged due to low sample sizes]), facility SatCom status (yes or no), state (Ondo, FCT, or Kano), and the number of days between FHWs’ pre- and posttests. The model also included a random intercept for individual FHWs to account for the repeated outcomes within individuals (ie, the pre- and posttest scores) and a separate random intercept for health facilities to account for any clustering effects at the health facility level. Using the fitted model, we then estimated the overall mean pre- and posttest scores, and the overall posttest minus pretest change in test scores (ie, the estimated change from before to after the intervention), along with the associated 95% CI and *P* values of these means. The means were based on estimated marginal means, also known as least-squares means or adjusted means, as calculated from the fitted model. The estimated marginal means assume a balanced population across all covariates, and when estimating them, we set the only numerical variable in the model (days between pre- and posttest) to its mean value across the sample.

We then estimated test score results within the following mutually exclusive sets of subgroups: (1) male or female FHWs; (2) CHEWs, or nurses and midwives; (3) FHWs in PHCs, or FHWs in comprehensive health centers or FHWs in health posts and basic health centers; (4) FHWs in facilities with SatCom available or FHWs in facilities without the availability of SatCom; and (5) FHWs based in facilities located in Ondo, FCT, or Kano states. To calculate these results, for each set of subgroups, we fitted the same multilevel linear regression model described above, but with an additional term for the interaction between the test period (pre or post) and the relevant categorical variable defining the relevant set of subgroups (eg, gender for male or female FHWs). Using each of these models, we then estimated the pre- and posttest scores and the posttest minus pretest change in the test score for each subgroup, along with the associated 95% CI and *P* values. Within each set of subgroups, we then explored whether the observed changes in test scores between the pre- and posttest periods differed between each subgroup (eg, male vs female FHWs). To do this, we used the same models with interaction terms described above to calculate the differences in estimated test score changes (from pre to post) between the relevant subgroups, taking one subgroup within each set as the reference or comparison group, along with their associated 95% CI and *P* values (again based on estimated marginal means). Finally, we also calculated adjusted overall posttest scores and their 95% CI for each separate topic covered by the test, by repeatedly fitting multilevel linear regression models with outcomes of each topic-specific posttest score, in turn, and independent variables and random intercepts that were the same as described above for the overall primary outcome analysis, excluding a variable for the test period.

For all analyses, we excluded observations (FHWs) if they were missing any outcome or required covariate data (ie, complete case analyses). We calculated CI and *P* values based on *t* statistics using the Kenward–Roger degrees of freedom approximation. We checked for adherence or violation of model assumptions using the standard range of residual and influence plots for multilevel linear regression models, but found no issues. All results were calculated using R version 3.5.2 statistical software (R Foundation for Statistical Computing) [22], with all models fitted using the *lme4* package [23], and all estimated marginal means calculated using the *emmeans* package [24].

### Qualitative Data Collection and Analysis

To assess the acceptability and feasibility of using the VTR mobile education intervention, we conducted face-to-face semistructured qualitative interviews with 34 participants in 3 states—12 FHWs, 12 facility managers, and 10 policy makers. Participants were recruited between February 19 and March 9, 2018. Interviews were conducted by 4 medical doctors (KO, AA, OD, and RMY) and a sociologist (DA), who were trained in qualitative interviewing techniques. Only 1 of the 5 data collectors was a female (OD). The research staff provided study information sheets to potential participants to help them understand the objectives of and decide whether to participate in the study. Participants were given at least 24 hours to express interest in participating in the study. Interview guides (Multimedia Appendix 2) were pretested before they were administered to the field. Interviews, which lasted about 30 minutes each, were conducted in a private setting in the workplace of respondents, audio-recorded, transcribed verbatim, and where appropriate, translated into English for analysis. The framework approach was used for analysis, while allowing for the emergence of new themes. The framework analysis involves the stages of familiarization with data, coding (done by the 5 interviewers above), indexing and charting, mapping, and interpretation [25]. The analysis was performed manually.

We drew on the technology acceptance model (TAM) to help explain stakeholders’ acceptance and use of VTR Mobile intervention in the workplace environment [26]. The TAM proposes that an individual’s acceptability of (ie, intent to use) and use behavior (ie, actual use) of a technology is determined by two variables. These are the perceived usefulness of the technology to enhance job performance, and the perceived ease of use of the technology, that is, the effort needed to learn and use a given technology. An individual’s motivation to use an emerging technology is higher if the technology is easy to use. The TAM also proposes that factors such as an individual’s understanding of a technology and organizational support measures have positive effects on the perception of usefulness and adoption of technology.

### Ethics Approval

Approval for the study was granted by the University of Leeds School of Medicine Research Ethics Committee (MREC16-178) and the Ondo State Government Ministry of Health (AD.4693 Vol. II/109), the Kano State Ministry of Health (MOH/Off/797/T1/350), and the Federal Capital Health Research Ethics Committee (FHREC/2017/01/42/12-05-17).

## Results

### Overview

We recruited and registered 349 FHWs for this study. However, 2.2% (8/349) of FHWs were doctors and did not complete the pretest. Of the remaining FHWs, 96.1% (328/341) completed both the pre- and posttest, and all had the necessary covariate data (Table 2). All pretests were completed between August 8, 2017, and March 16, 2018, in Ondo state; between August 10, 2017, and February 21, 2018, in Kano state; and between July 10, 2017, and March 15, 2018, in FCT, whereas all posttests were completed between February 23, 2018, and May 21, 2018, in Ondo state; between March 7, 2018, and May 2, 2018, in Kano state; and between March 5, 2018, and April 30, 2018, in FCT. After taking their pretest, FHWs took a mean of 152 days to complete their posttest; however, this varied substantially, ranging from 2 to 279 days (IQR 138.75; Table 3). More specifically, 28.3% (93/328) completed in 4 to 90 days, 17.9% (59/328) completed in 91 to 180 days, and 53.6% (176/328) completed in 181 to 279 days. This result highlights that most FHWs completed their posttest in the final 3 months of the (approximately) 9-month period during which all posttests were completed. Recruited FHWs were primarily female CHEWs based within PHCs and comprehensive health centers, but because comprehensive health centers contain multiple FHWs (mean 12.2), HPs typically contain only 1 or 2 FHWs (mean 1.1), and there were approximately 3 times more HPs than CHCs in the study (Tables 3 and 4).

Less than one-third of all FHWs accessed eHealth materials via SatCom, and SatCom access was available for all health posts and basic health centers in the study, whereas availability was nearly evenly split for PHCs and CHCs (Table 4). Just over half of the FHWs (180/328, 54.9%) were based in facilities in Ondo state, just over a third (115/328, 35.1%) were based in the FCT state, and 10% (33/328) were based in the Kano state (Table 3).

**Table 2:** Overview of recruitment and the completion of pre- and posttests by frontline health worker staff type (N=349).

	Pretest completion, n (%)	Posttest completion, n (%)
Doctors (n=8)	0 (0)^a^	0 (0)
Nurses and midwives (n=31)	31 (100)	31 (100)
CHEWs^b^ (n=310)	297 (95.8)	297 (95.8)
Total (N=349)	328 (93.9)	328 (93.9)

^a^A total of 8 persons were excluded from the analysis due to incomplete tests.

^b^CHEW: community health extension worker.

**Table 3:** Frontline health worker characteristics (N=328).

Characteristics			Values
**Gender, n (%)**			
	Female	220 (67)	
	Male	108 (32.9)	
**Cadre, n (%)**			
	CHEW^a^	297 (90.5)	
	Nurses and midwives	31 (9.4)	
**Type of facility, n (%)**			
	Primary health center	235 (71.6)	
	Comprehensive health center	61 (18.5)	
	Health post	25 (7.6)	
	Basic health center	7 (2.1)	
**SatCom^b^ available at facility, n (%)**			
	No	233 (71)	
	Yes	95 (28.9)	
**Facility location, n (%)**			
	Ondo	180 (54.9)	
	FCT^c^	115 (35.1)	
	Kano	33 (10)	
Days between pretest and posttest, mean (SD)			152.7 (82.1)

^a^CHEW: community health extension worker.

^b^SatCom: satellite communication (including internet).

^C^FCT: Federal Capital Territory.

**Table 4:** Facility characteristics (N=138).

Facility type	Mode of delivery of eHealth interventions, n (%)			Facility type total, n (%)
	SatCom^a^ sites^b^	Non-SatCom sites^c^		
Primary health center	40 (28.9)	59 (42.7)	99 (71.7)	
Health post	25 (18.1)	0 (0)	25 (18.1)	
Comprehensive health center	4 (2.8)	4 (2.8)	8 (5.7)	
Basic health center	6 (4.3)	0 (0)	6 (4.3)	

^a^SatCom: satellite communication (including internet).

^b^Total satellite communication sites: 54.3% (75/138).

^c^Total satellite communication sites: 45.6% (63/138).

Overall, FHWs achieved a mean pretest score of 51% (95% CI 48%-54%) and a mean posttest score of 69% (95% CI 66%-72%), and after adjusting for key covariates, this represented an overall mean increase in test score between the pre- and posttest of 17 percentage points (95% CI 15-19; *P*<.001; Table 5). There was an indication that male FHWs’ test scores increased slightly less on average than female FHWs (−5 percentage points, 95% CI −9 to 0; *P*=.03), and a much clearer indication that FHWs in Ondo state increased their test scores much more on average than FHWs in Kano state (9 percentage points, 95% CI 3-16; *P*=.005; Table 5). However, there were no clear differences in the observed changes in test scores between different types of FHWs, FHWs in different types of facilities, or FHWs in facilities with and without SatCom availability.

**Table 5:** Overall subgroup-specific and between-subgroup estimates of frontline health workers’ mean pre- and posttest scores and mean pretest to posttest change in test scores^a^.

Characteristics		Value, n (%)	Pretest score (95% CI; %)	Posttest score (95% CI; %)	Pre- to posttest scores		Between-subgroup difference in pre- to posttest scores	
					Point change (95% CI; %)	*P* value	Point change (95% CI; %)	*P* value
Overall		328 (100)	51 (48 to 54)	69 (66 to 72)	17 (15 to 19)	<.001	N/A^b^	N/A
**Staff sex**								
	Male	108 (32.9)	52 (48 to 55)	66 (62 to 69)	14 (11 to 18)	<.001	−5 (−9 to 0)	.03
	Female	220 (67)	52 (48 to 55)	71 (67 to 74)	19 (16 to 21)	<.001	Reference	N/A
**Staff type**								
	CHEW^c^	297 (90.5)	49 (46 to 52)	66 (63 to 69)	17 (15 to 19)	<.001	−1 (−8 to 6)	.75
	Nurses and midwives	31 (9.4)	54 (48 to 59)	72 (67 to 77)	18 (12 to 25)	<.001	Reference	N/A
**Facility type**								
	PHC^d^	235 (71.6)	53 (49 to 57)	70 (66 to 74)	17 (15 to 19)	<.001	2 (−4 to 9)	.50
	Comprehensive health center	61 (18.5)	50 (43 to 57)	70 (63 to 78)	21 (16 to 25)	<.001	6 (−2 to 14)	.13
	Health posts and basic health centers	32 (9.7)	51 (43 to 59)	66 (58 to 73)	15 (8 to 21)	<.001	Reference	N/A
**SatCom^e^**								
	Yes	95 (28.9)	51 (47 to 55)	67 (63 to 71)	16 (12 to 19)	<.001	−2 (−7 to 2)	.29
	No	233 (71)	52 (48 to 56)	70 (67 to 74)	18 (16 to 20)	<.001	Reference	N/A
**State**								
	Ondo	180 (54.8)	53 (48 to 58)	76 (71 to 80)	23 (20 to 25)	<.001	9 (3 to 16)	.005
	FCT^f^	115 (35)	56 (51 to 62)	67 (61 to 72)	10 (7 to 13)	<.001	−3 (−10 to 4)	.39
	Kano	33 (10)	48 (40 to 56)	61 (53 to 69)	13 (7 to 19)	<.001	Reference	N/A

^a^All values are based on estimated marginal means calculated from multilevel linear regression models. All models have an outcome of frontline health workers’ (FHWs’) pre- and posttest score values, which were measured as the percentage of correct answers on a 48-question multiple-choice test of FHW knowledge, attitudes, and reported practices with respect to maternal, newborn, and child health. All models include independent variables for FHWs’ sex, FHWs’ staff type (community health extension workers or nurses and midwives), FHWs’ facility type (primary health care, comprehensive health center or health posts and basic health centers), FHWs’ facility SatCom status (yes/no), FHWs’ facility location (Ondo, Federal Capital Territory, or Kano), and the number of days between FHWs’ pre- and posttests. All models also include random intercepts for FHW and facility to account for clustering of pre- and posttest outcome scores within FHWs and facilities. All CIs and *P* values are based on *t* statistics using the Kenward–Roger degrees of freedom approximation. Any FHWs with missing outcome or covariate data were excluded. Refer to the *Methods* section for full details.

^b^N/A: not applicable.

^c^CHEW: community health extension worker.

^d^PHC: primary health care.

^e^SatCom: satellite communication.

^f^FCT: Federal Capital Territory.

Our analysis of the topic-specific test scores showed that, on average, FHWs appeared to do worse on questions about warning signs in pregnancy (topic 2 in Table 6), prevention and management of postpartum hemorrhage (topic 5), and neonatal resuscitation (topic 8) compared with questions on all other topics, but better on questions on respectful maternity care (topic 2) and how to care for a newborn (topic 9) compared with questions on all other topics.

**Table 6:** Overall frontline health workers’ topic-specific posttest scores based on the percentage of correct answers to questions on specific topics on maternal and child health care knowledge and reported attitudes and practices.

Topic number^a^ and description	Scores (95% CI)
1. Focused antenatal care	63 (60-65)
2. Respectful maternity care	84 (82-87)
3. Warning signs in pregnancy	51 (49-54)
4. How to use a partograph	67 (65-70)
5. Prevention of PPH^b^	53 (51-56)
6. Management of PPH in a low-resource setting	65 (63-68)
7. Manual removal of placenta	66 (64-69)
8. Neonatal resuscitation	50 (47-52)
9. How to care for a newborn	78 (76-79)

^a^Topic numbers refer to the order in which topics were asked in the test.

^b^PPH: postpartum hemorrhage.

### Characteristics of Stakeholders Interviewed

A total of 34 stakeholders were interviewed in 3 states regarding the acceptability, feasibility, and use of the VTR Mobile intervention (Table 7). Approximately 35% (12/34) of respondents (stakeholders) interviewed were FHWs. Another 35% (12/34) of respondents were health facility managers, whereas the remaining 29% (10/34) were policy makers.

**Table 7:** Characteristics of respondents by stakeholder group interviewed (N=34).

Respondent group	Respondents, n (%)			
	FCT^a,b^	Kano state^c^	Ondo state^d^	Total^e^
FHW^f^	4 (11)	4 (11)	4 (11)	12 (35)
Facility managers	4 (11)	4 (11)	4 (11)	12 (35)
Policymakers	3 (8)	3 (8)	4 (11)	10 (35)

^a^FCT: Federal Capital Territory.

^b^Total respondents in Federal Capital Territory: 32% (11/34).

^c^Total respondents in Kano state: 32% (11/34).

^d^Total respondents in Ondo state: 35% (12/34).

^e^Total respondents interviewed: 100% (34/34).

^f^FHW: frontline health worker.

### Qualitative Findings: Factors Affecting the Acceptance and Use of VTR Technology

Findings from IDIs with FHWs, facility managers, and policy makers showed a wide acceptance of VTR mobile technology as an important tool for enhancing the quality of training for health workers and the standard of health care delivery. Stakeholders described the introduction of VTR Mobile as highly beneficial for FHWs in Nigeria and cited the following five drivers of acceptance of tablet-optimized VTR: (1) perceived ease of use of VTR Mobile app and platform; (2) accessibility to tablet computers; (3) convenience of offline access to training content, making videos reliable reference materials; (4) perceived usefulness of training content for improving life-saving skills; and (5) cost-effectiveness of VTR. As respondents often referred to two or three drivers of VTR mobile acceptance in their responses, the quotations outlined supporting drivers (detailed in Table 8) may allude to more than one determinant of acceptability in the same extract.

Despite the barriers outlined above, participants reflected views that the VTR was a promising means for (1) providing high-quality training in a cost-effective way; (2) reinforcing knowledge, enhancing skills, and increasing FHW confidence; (3) improving health care–seeking behavior among women; and (4) reducing maternal and infant mortality and morbidity.

**Table 8:** Key themes of the acceptability and feasibility of using VTR^a^ Mobile with supporting quotes.

Determinants of acceptability and feasibility and description of key findings			Supporting participant quote
**Ease of use of VTR Mobile is linked to perceived accessibility**			
	Facility managers and FHWs^b^ found it easy to use the technology. Respondents highlighted how access to tablet computers in participating health facilities made it easy to use the VTR Mobile App and, by doing so, increased acceptability of the intervention.	“Having this video and tablet here [in the facility] contributes enough [to making us use it] and the videos give us more guidance on how to manage some minor illnesses that we didn’t have more knowledge on them before.... So it is a welcome development.” [Facility Manager, Gwagwalada, FCT^c^]	
	The feasibility of using tablet computers and the VTR Mobile app was aided by the introductory training provided at the beginning of the study and ongoing support to solve technical problems that arose during the use of devices. The training increased familiarity with and use of digital devices and VTR Mobile app. The link between accessibility to devices and motivation to use the training app in the above quote is supported by a policy maker, who also underlined the prospect of tracking technology for use in monitoring completion and noncompletion of pre- and postintervention tests.	“The use of VTR has been more regular and on ground. It is regular [because when you have the tablet], you can watch the videos as many times as you want, countless times...you can just open it and watch.... But they [InStrat] have their own assessment too, we also check with them [InStrat] to know how many [participants] are watching the contents. But the assessment you do, they are a kind of mini exam, we will know who are watching and applying what they have learnt from it.” [Policy Maker, Gwagwalada, FCT]	
**Training videos as aide-mémoire for clinical practice**			
	Once the videos are downloaded from the VTR Mobile platform onto tablet computers, the training content can be watched repeatedly (offline use) at no additional cost to FHWs and facility managers. The convenience of repeated use of the videos has made them a reliable reference material for FHWs. It was common for FHWs to gather in groups to watch the videos. They found the video-viewing sessions and their associated vignettes helpful for knowledge exchange, problem-solving, and peer support.	“I think some staff are beginning to look at this thing [clinical videos on tablets] as an important, err, aspect of work. The video helps us...it makes the work easier. If we are in any difficulty, we just go to that particular video and watch it many times. And now, we know what to do for clients.” [Facility manager in Gwagwalada, FCT]	
**Perceived usefulness of VTR Mobile for improving service provision**			
	A key driver of acceptability cited by FHWs was the perceived usefulness of VTR Mobile for improving the quality of health care provision. Participant narratives demonstrated the utility of computer-optimized videos for increasing knowledge, clarifying areas of confusion, boosting FHW capability to manage birth-related complications and preventing infant mortality. Although we expected the integration of VTR Mobile into routine health care practice to increase FHW knowledge and confidence to deliver high-quality life-saving care, an unanticipated finding was that access to VTR Mobile empowered FHWs to use the videos to conduct health education sessions for women attending ANC^d^. This unplanned use of VTR Mobile inspired pregnant women to broadcast the availability of new technology at intervention facilities in the community, leading to increased attendance at ANC classes (ie, improved health care–seeking behavior). Participants described how VTR Mobile also led to improved attitudes of FHWs toward patients, which in turn increased service users’ confidence in FHWs, which subsequently increased FHW confidence to provide respectful and high-quality care.	“You know sometimes when you don’t know something [about a topic], and when you continue to receive cases related to that topic, you will be feeling demoralized and doubtful, because you don’t know what to do. But when you already have the solution in your hands [as we now do with VTR], then, you will be very confident in what you do, and in the answers, you provide to patients.” [FHW in Onikokodiya, Ondo state] “The last time we used the tablet [to watch videos], it helped us to resuscitate a newborn baby. Before now, when a baby was born, and the baby was not breathing normally, we used to do the mouth-to-mouth [resuscitation] but...our attempts did not work because of this tab on the Ambu bag. This time around, when we had a baby that had trouble breathing, we opened the computer tablet and watched the video, and immediately we picked up our Ambu bag and used it as was shown in the video and the baby began to breath normally again.” [Facility manager in Aseigbo, Ondo] “VTR has really helped a lot because attitude of staff towards patient is one of the most important aspects of healthcare delivery because if you don't show your good conduct, it's like you are driving away your clients. But the more you are closer to patients, the friendlier you are with them, the more they will come to the health facility. They will have more confidence in you, and they will tell you more about themselves and it will help in treatment and other services that you want to provide. Patient-staff relationship is particularly important because that's where confidence will develop. That'll make your work even easier and better because they will not hide anything [from you]. They will tell you the truth and it help you in your diagnosis and even in standard of your work too.” [FHW in the FCT]	
**Cost-effectiveness of VTR**			
	What constitutes usefulness, however, may be different across different stakeholder groups depending on whether respondents were health workers or policy makers. Although FHWs and facility managers regarded improved knowledge, skills, and confidence as benefits of VTR, policy makers, in contrast, saw tablet computers loaded with VTR Mobile as a cost-effective intervention for reducing the cost of conventional training of FHWs.	“In my own assessment, VTR Mobile has been awesome and the experience is encouraging in the sense that health care staff will not need to travel and go anywhere [for training]. Training materials are now available with them [on tablets in health facilities]. While visiting a facility in a very remote area, I heard a testimony from a staff who had a patient with PPH [postpartum hemorrhage] and how she had watched the video clip on the training to be able to manage PPH. She came out [of the labor room] after helping the patient and told us what she did in line with what the video directed her to do. It was an awesome experience...when I heard it, I was happy.” [Policymaker in Gwagwalada, FCT]	
	Policy makers seemingly found the experience of using clinical videos to improve health care provision in rural areas particularly intriguing, with the potential for mobile technology to improve primary health care. Policy makers believed that the access to VTR Mobile provided an opportunity to substantially reduce the cost of training while allowing the government to train more FHWs despite several competing priorities. The average cost of training fell by 79.6% to US $509 per year in the project intervention sites compared with US $2489 per year for face-to-face training of CHEWs^e^ in Sub-Saharan Africa [27]. The figure of US $509 per year includes the cost of delivering 40-hours of VTR content, supplying hardware, and providing technical support to FHWs.	“Very well it [VTR] is improving health care positively...I go round like these places [visiting facilities], and the testimonies or reports coming from all sites, particularly the hard-to-reach areas [are remarkable]....These are places where there are no ambulances to convey anybody. Not even motorcycle to transport anybody to the nearest clinic [or hospital]. I am convinced you can strengthen Primary Health care through this project...if we can strengthen Primary Health care [using VTR], other aspects of health care will get this right too.” [Policy Maker, Gwagwalada, FCT]	
**External barriers to the use of VTR**			
	Despite the reported positivity toward VTR, three external (structural) barriers affected the adoption and use of VTR: (1) poor internet connection to log into the VTR Mobile platform, (2) poor electricity supply to charge devices, and (3) workload issues that prevented FHWs from completing pre- and posttests. Regarding internet connectivity and electricity supply, a minority of respondents reported how they temporarily stopped using VTR Mobile due, in part, to the lack of electricity to power the tablets and the lack of internet connectivity (previously enabled through satellite communication technology). Participants were unable to charge devices when rechargeable solar batteries installed at each participating health facility were drained and flat and went outside the facility to charge the device at a nominal cost.	“Like that of tablet, there was a problem of network, and that of electricity that you are talking about. You know, there is no NEPA [referring to National Electric Power Authority, the former name of an organization governing the use of electricity in Nigeria] supply [ie, mains electricity] to the facility. So if you want to watch the video, you either charge the tablet, [and as there’s not mains electricity], or they [the staff] will carry the tablet outside there [to the nearby town] and then I give them money [to pay to charge the device].” [Facility manager in FCT]	
	A few health workers reported that the workload at the facility level prevented them from watching all VTR Mobile videos and completing the pre- and posttest promptly. However, it is unclear what proportion of late completers of pre- and posttests referred to in the quantitative section of this paper were affected by clinic workload and tight schedules. Policy makers outlined efforts to address workload issues through providing alternative access to the VTR Mobile app, for example, by supporting FHWs to install the app on personal Android phones to facilitate self-study at home.	“[For FHWs struggling to complete the tests]...they [InStrat] have made it so easy that they can upload VTR on their phones [of FHWs]. During the pilot study, we encouraged them [FHWs] to get it. Those who have android phones that they can have it on their phones, so that it is not only when they get to the health facility where they have only one tablet that they can do it.” [Policymaker in Ondo state]	

^a^VTR: video training.

^b^FHW: frontline health worker.

^c^FCT: Federal Capital Territory.

^d^ANC: antenatal care.

^e^CHEW: community health extension worker.

## Discussion

### Principal Findings

Although digital health approaches have shown promise in improving health care provision in LMICs, they are infrequently implemented at scale [28]. This study focused on understanding the acceptability, feasibility, and potential effectiveness of an e-learning video–based intervention on MNCH transmitted at scale to FHWs in rural areas of Nigeria. We found that following the use of the e-learning intervention, FHWs demonstrated substantial improvements in their scores on a test of their knowledge, attitudes, and reported practices about MNCH, indicating that the e-learning intervention is potentially effective in improving knowledge, attitudes, and practices in this area. Using the TAM to guide qualitative data analysis, we also identified five determinants of acceptance and three barriers to the use of VTR Mobile for training FHWs.

The five determinants of acceptance highlighted in the findings of this study were (1) the perceived ease of use of technology, (2) the perceived usefulness of clinical videos to enhance job performance, (3) access to tablet computers in the workplace, (4) the convenience of offline access and repeated use of training content, and (5) the perceived cost-effectiveness of VTR for FHW training. The latter three determinants of acceptance in Nigeria help to extend the classic TAM, which often prioritizes the perceived ease of use and usefulness of technologies as principal factors of acceptance. Qualitative data analysis showed how contextual factors such as previous training of FHWs to understand the e-learning technology, organizational support factors such as access to technology in the workplace, and technical support during the e-learning intervention increased the acceptance of and FHW confidence in using VTR to improve service delivery. In addition, our data also revealed how the convenience of offline access to training content at no cost to FHWs combined with the perceived usefulness of VTR to improve FHWs’ knowledge and attitudes toward patients seemingly sparked the habitual use of clinical videos as reference material to guide live-saving procedures, which in turn increased FHW confidence to provide high-quality care. Furthermore, insight from Table 8 highlights how improved staff attitudes toward patients stimulated confidence in FHWs, which apparently generated a virtuous circle of increased FHW confidence to provide respectful care leading to service user confidence in FHWs and improved staff-patient relationships that subsequently boosted FHWs’ confidence in providing respectful care. Conversely, three barriers that constrained the adoption and use of VTR Mobile in Nigeria were external factors that were the downstream of VTR technology. These structural factors were as follows: first, poor internet connection in a few health facilities served by the 3G mobile networks prevented FHWs from logging into the VTR Mobile platform, thereby limiting access to the training content. It is important to emphasize that accessibility to the internet was not constrained by affordability issues, as tablet computers used by FHWs were loaded with prepaid data plans to ensure seamless access to the e-learning platform. Second, poor electricity supply affected FHWs’ ability to charge tablet computers mainly in health facilities located in rural areas and in facilities with empty and uncharged solar batteries. Third, organizational workload issues arising from technology introduction into the workflows of primary health centers limited FHWs’ ability to complete the pre- and posttest surveys in some facilities. Taken together, the foregoing five determinants of acceptance and three barriers to the adoption and use of VTR broadly affected the effectiveness of the e-learning intervention in Nigeria.

### Comparison With Previous Work

The distinctive features of this study are its identification of evidence of the potential effectiveness and feasibility of deploying digital health approaches for improving the knowledge, attitudes, and practices of FHWs at scale and to explain the possible mechanisms of the impact of VTR on staff performance and health system functions (service delivery and financing) in an LMIC. This study also addresses earlier calls for studies with larger sample sizes, more quantitative methods of evaluation, and exploration of implementation at scale while assessing the effectiveness of digital health approaches to train health care professionals in resource-limited countries [29-32]. The overall findings of our research align with those of previous digital health research in which multimedia content was delivered at scale to FHWs in Nigeria and other LMICs and which found that (1) FHWs had positive attitudes toward technology-enabled learning; (2) previous training and familiarity with technology increased usability; (3) digital health approaches were potentially effective for increasing FHWs’ knowledge, attitudes, and care practices; and (4) digital health approaches also empowered workers with skills and confidence in contexts where technology adoption enhanced their performance and supported their work [14,29,30,33]. These findings have implications for developing strategies that ensure adequate orientation and continuing technical support for FHWs to adopt and use digital technology to achieve individual and organizational goals. Furthermore, these findings increase the evidence base underpinning the potential effectiveness of digital provision of training and educational content for health workers, which has previously lacked evidence to inform health workers’ performance, skills, and attitudes [4].

Most digital health interventions to support task-shifting and community health worker (CHW) training in LMICs [34] used smartphones and basic feature phones [35]. Only a few e-learning interventions have adopted tablet-based apps [34,36]. However, evaluations of digital health projects to scale up CHW training in Pakistan [36] and India [37] have reported effective training interventions that increase CHW knowledge, motivation, and competence. For example, the *Sangoshthi* project, which scaled up CHW training to benefit more than 900,000 CHWs across India [37] recorded knowledge gains of 16% between pre- and posttest assessments. This is comparable with our findings from Nigeria, which showed a knowledge change of 17% between pre- and posttest scores; however, the *Sangoshthi* project was silent about the effects of its intervention on patient outcomes (micro or individual level), service delivery (meso or organizational level), and policy decisions (macro or wider system). In contrast, our study suggested that delivering video-based training at a scale can positively impact the micro, meso, and macro levels of the health system. The analysis of qualitative data showed that at the micro level, access to tablet devices, training opportunities, and ongoing technical support can increase staff confidence and motivation. At the meso level, better trained and skilled staff at intervention facilities felt empowered to deliver improved services, which manifested as respectful care, better management of complications, and enhanced patient engagement activities during antenatal clinic classes. This suggests that organizational contexts that provide essential equipment (in this case, tablet devices and clinical videos) to support the work of frontline staff can empower them to improve their performance. This further suggests that institutional readiness in human and infrastructural resources is necessary for e-learning; however, it is not always present in LMICs [38]. At the meso level, we also found that the availability of clinical videos that incorporated vignettes with relevant questions sparked team-level discussions that provided opportunities for problem-solving, knowledge-sharing, and peer support. Finally, at the macro level, we saw how implementing a digital health approach to training can influence strategic policy decisions about which model of training to invest in scarce resources.

It is vital to underline that the macrolevel support was crucial for the benefits and impacts realized at the micro and meso levels of this project. A policy environment that enabled digital health care to function in the three states facilitated the successful adoption and implementation of SatCom and VTR mobile technologies. State-level ICT policies were aligned with the Federal Government of Nigeria’s prioritization of ICTs as a strategy for achieving universal access to essential PHC services for mothers and their children [9]. Furthermore, this project is an example of thriving public-private partnerships between the government and local technology companies to develop digital health initiatives in Sub-Saharan Africa with initial financial inputs, technical know-how, and operational efficiency to ensure long-term adoption of digital health care. Such private sector involvement is essential for sustainable implementation if universal health coverage is achieved in Nigeria. Although the approach described in this paper has been successful, it relied on the use of SatCom technology alongside existing 3G mobile networks to overcome telecommunications challenges, as more than half of PHC facilities located in rural areas lacked internet connectivity. This further highlights the need to include the development of critical ICT infrastructure as a strategy to increase the quality of health care delivery in LMICs. Facilitating uninterrupted access to data and networks is a prerequisite to delivering approaches such as VTR Mobile, which have clear benefits for FHWs and recipients of their care. The application of space technologies (eg, SatCom) as used to overcome these challenges in this project required building partnerships with commercial partners. Although there are increasing examples of the application of space technologies such as SatCom technology in global health research, there is a need for improved awareness, training, and collaboration of the research community in such endeavors [39]. We have demonstrated the potential of PHCs to access education resources in the context of this project and suggest this as a priority area for digital health research in the context of LMICs.

### Limitations

Although we provide evidence indicating the potential effectiveness of the intervention, our quantitative study design has several important limitations. First, an uncontrolled before-and-after comparison lacks the robustness and comparability of a randomized experimental design [40], which could compare an e-learning intervention to conventional face-to-face training. More specifically, uncontrolled before-and-after studies face threats to internal validity because of several possible biases. These include regression-to-the-mean bias, maturation bias (ie, changes in participants’ cognitive abilities due to aging), attrition or loss to follow-up bias, retrospective bias, history bias (sometimes referred to as *secular effects*), and test-retest bias. We do not believe that our study was at risk of any substantial regression-to-the-mean bias because we did not restrict our recruitment of health workers based on any characteristics, but allowed all health workers within a selected facility to participate if they wished. It is also unlikely that our study would have experienced much maturation bias due to the relatively short time between the before-and-after comparisons. We also do not believe that we suffered any substantial attrition bias due to our high level of follow-up (328/341, 96.1%), and we avoided any retrospective bias by using a prospective design. We also believe that the duration of time between completion of pre- and posttest scores reduces the likelihood of history bias, with a limited opportunity for external initiatives to have influenced changes in participant scores. It is likely that our study suffered from test-retest bias, which implies that an unknown amount of the observed increase in test scores is probably due to participants remembering their pretest errors and correcting them, rather than having improved their knowledge via the intervention. Therefore, collectively, these risks must be considered while interpreting the results. Second, we only used pre- and posttest assessments of responses to questions on knowledge, attitudes, and reported practices related to MNCH to assess the effectiveness of the intervention. More objective and rigorous measures of performance and appropriate care behaviors would enhance the robustness of this or future evaluation. Third, although interview questions were developed and refined with key stakeholders and interviews conducted by experienced researchers, interview guides were not pilot-tested before use. Interview guides clarified the intended data to be derived from questions, but pilot testing may have refined the items used and improved the richness of data received from participants. Fourth, evaluation at multiple follow-up periods would provide useful information on the longevity of any intervention effects. Finally, the average cost of training described in this project excludes the cost of the SatCom component of the project, which made the project unsustainable for state governments in Nigeria. We did not evaluate the cost-effectiveness of the intervention as part of this study, which should be explored in future studies. However, the qualitative component did highlight that policy makers perceive tablet computers loaded with VTR Mobile as a means of reducing the costs associated with conventional training of FHWs.

### Conclusions

This is the first report of combining SatCom with existing 3G mobile networks to support the VTR of FHWs at scale in LMICs. The study showed a widespread acceptance of VTR among FHWs with five determinants of acceptance in Nigeria: ease of use, perceived usefulness of VTR for improving service delivery, access to tablet computers in the workplace, convenience of offline access to training contents, and cost-effectiveness of VTR. The evaluation also demonstrated the potential effectiveness of the e-learning intervention in improving the knowledge, attitudes, and confidence of rural FHWs. It also identified the possible mechanisms of the impact of this e-learning approach at the micro, meso, and macro levels of the health system. Nonetheless, it raises questions about structural barriers to VTR adoption and use in areas that lack internet connectivity, experience poor electricity supply, and increased FHW workloads arising from technology introduction. Policy strategies for improving workforce performance should create working environments that provide critical infrastructure to ensure an uninterrupted electricity supply and internet connectivity that support sustained access to reliable training content and enable workers to apply their knowledge and skills to deliver respectful and high-quality health care that promotes UHC.

## Appendix

Multimedia Appendix 1 Items for pre- and posttest.

Multimedia Appendix 2 Topic guide.

## Abbreviations

CHEW: community health extension worker
CHW: community health worker
FCT: Federal Capital Territory
FHW: frontline health worker
ICT: information and communications technology
IDI: in-depth interview
LMIC: low- and middle-income country
MNCH: maternal, newborn, and child health
PHC: primary health care
SatCom: satellite communication
TAM: technology acceptance model
VTR: video training
WHO: World Health Organization
