# Supplementary material for: Acceptability and Potential Effectiveness of eHealth Tools for Training Primary Health Workers From Nigeria at Scale: Mixed Methods, Uncontrolled Before-and-After Study
Source: JMIR Mhealth Uhealth. 2021 Sep 16;9(9):e24182. doi: 10.2196/24182 (PMC8485189; doi:10.2196/24182)
Supplement: Multimedia Appendix 1 [file mhealth_v9i9e24182_app1.docx]

**Multimedia Appendix 1:**

**Test questions administered for pre- and post-test of the Video Training Intervention**

| **Topic Areas** | **Questions asked** | **Multiple choice options** | **Answer** |
| --- | --- | --- | --- |
| **Importance of Focused Antenatal Care** | 1. What are your jobs/tasks during focused antenatal care visits | A. Warn mother about dangers of pregnancy B. Observe and act on risk factors C. Show pregnant mother you know best D. Do as much paperwork as possible | B |
|  | 2. When should the four standard focused antenatal care visits be made? | A. Around 26 weeks of pregnancy B. Every 2 weeks C. Whenever you are visiting someone in her village D. When the woman feels like it | A |
|  | 3. What examination should you carry out at each focused antenatal care visit? | A. Take blood pressure of pregnant mother  B. Measure height C. Measure Shoe size D. Note eye colour | A |
|  | 4. What other examinations should you make at each focused antenatal care visit? | A. Confirm pregnancy B. Note eye colour C. Estimate due date D. Look for warning signs early on | B |
|  | 5. What TWO should you remember at the second focused antenatal care visit? | A. Carry out routine checks B. Give religious guidance C. Document this visit D. If all is well, tell her not to come back | A and C |
| **Respectful Maternity Care** | 1. Who else might Mama (the pregnant mother) not want with her at the birth? | A. Sister B. Friend C. Boss D. Husband | C |
|  | 2. How could you give Mama (the pregnant mother) privacy during birth? | A. Use a folding screen to provide privacy  B. Introduce Mama (pregnant mother) to everybody in the health Centre C. Encourage her to share a bed | A |
| **Warning Signs in Pregnancy** | 1. What are the signs of anaemia? (SELECT TWO) | A. Baby moving a lot B. Breathlessness C. Low blood pressure d. Yellow eyeball E. Fast heartbeat | B and E |
|  | 2. What CANNOT be done to help manage anaemia? | A. Give Iron B. Seek help C. Send expectant mother to hospital D. Improve diet E. Encourage Spacing of pregnancies | C |
|  | 3. What is the most likely cause of an expectant mother bleeding? | A. Placenta is covering cervix B. Pregnancy is over C. Baby is bleeding | A |
|  | 4. What are the warning signs of pre-eclampsia? (SELECT TWO) | A. Swollen feet, ankles, hands and face B. Skin rash C. Headaches D. Pale eyelids E. Bleeding | A and C |
|  | 5. What are the signs of possible infection in a pregnant mother? (SELECT TWO) | A. Skin hot to touch B. Bleeding C. Low blood pressure D. Sweating E. Pale skin on palms of hands | A and D |
|  | 6. What can observations of the pregnancy bump NOT tell you? (SELECT TWO) | A. Likelihood of obstructed labour B. Eye colour C. Position of babies in the uterus D. How many babies in the womb  E. Intelligence of babies | B and E |
| **How to use a partograph** | 1. How can a simple partograph chart save lives? | A. The partograph stays with expectant mother even after health workers change shifts to allow other staff to see any danger signs B. The chart is responsible for saving lives, not the staff c. The chart tells you what to do in an emergency D. Paperwork is always good for its own sake | A |
|  | 2. What should you remember about using partographs? | A. Senior health worker is responsible B. New chart every shift C. Guess readings if you missed an observation D. Have blank copies ready in advance in case the one being used is full | D |
|  | 3. Which is NOT among the three vital observations, to track progress of labour, using a partograph | A. Dilation of cervix B. Height of mother C. Descent of baby's head D. Time in active labour | B |
|  | 4. Which is NOT among the THREE observations you record on the partograph, to track the health if the baby? | A. Foetal heart rate B. Moulding of baby's head C. Temperature of room D. Colour of amniotic fluid | C |
|  | 5. Which observations do you NOT record on the partograph about the condition of the mother herself? | A. Pulse and blood pressure B. Frequency of contractions C. Strength of the mother’s voice D. Contraction rate and strength E. Drugs administered | C |
| **Prevention and Management of PPH** | 1. In prevention of PPH, Ergometrin can be used rectally? | A. True B. False | F |
|  | 2. In the treatment of PPH, 40 units of oxytocin can be safely administered in 5% 500ml dextrose water solution | A. True B. False | F |
|  | 3. Uterine tamponade can take the place of Bi- manual compression to control uterine bleeding | A. True B. False | T |
|  | 4. Misoprostol can be used intra-vaginally to prevent PPH | A. True B. False | F |
|  | 5. Misoprostol is the drug of choice in the treatment of PPH | A. True B. False | F |
| **management of PPH in a Low resource setting** | 1. It is easy to predict when a woman will develop PPH | A. True B. False | F |
|  | 2. Early breast feeding always prevent PPH | A. True B. False | F |
|  | 3. A full bladder after delivery can cause PPH | A. True B. False | T |
|  | 4. What is a post-partum haemorrhage? | A. An anaemic woman losing more than 300ml (can of coke volume) of blood after delivery B. Any bleeding during delivery C. Any bleeding before childbirth D. healthy woman losing more than 400ml of blood after delivery | A |
|  | 5. Who is NOT at risk from post-partum haemorrhage? | A. women giving birth to more than one baby B. After prolonged labour C. Women giving brith for the fifth time or more D. Only mothers over 30 are at risk | D |
|  | 6. How can you actively manage the birth to reduce risk of postpartum haemorrhage? | A. Cut any mother to facilitate birth B. Avoid unnecessary episiotomy C. Pull hard on cord to bring placenta down quickly D. Leave mother with baby immediately after birth | B |
|  | 7. Where can trauma NOT cause excessive bleeding after birth? | A. Fistula with rectum b. Vaginal tears c. rupture of uterus itself D. Normal child birth can result in massive bleeding | D |
|  | 8. When can post-partum haemorrhage happen? | A. PPH is always fatal B. PPH is very rare and not serious C. Primary PPH happen within hours of delivery D. PPH is only of concern immediately after birth | C |
| **Manual removal of the placenta** | 1. How can you NOT promote a natural delivery of the placenta? | A. Ask mother to walk around B. Pull on cord while protecting fundus C. Encourage emptying bladder D. Encourage breastfeeding or stimulate nipples | A |
|  | 2. It is not compulsory to empty bladder | A. True B. False | F |
|  | 3. Antibiotics may not be needed if you wear gloves | A. True B. False | F |
|  | 4. What needs to be done before a manual removal of the placenta | A. Record baby's heart rate b. Measure and weigh patient C. Give painkillers to control pain D. Take baby away | C |
|  | 5. What are the TWO key points for patient's well-being during manual removal of the placenta? | A. Remove entire placenta even if it has broken up B. repair any tears c. Remove the biggest section of the placenta then leave the patient in peace | A and B |
| **Neonatal Resuscitation** | 1. Select TWO key points you have learned about resuscitation of a newborn baby | A. Get the heart going even if baby is not breathing B. For breathing problems, open the airway C. Birth is the main thing D. The first minute is crucial, so assess baby right away E. Make it up as you go | B and D |
|  | 2. When would you decide to stop unsuccessful resuscitation of a newborn baby? | A. Never give up B. Have an agreement with your team about when to stop C. After several hours D. After 3 or 4 minutes | B |
|  | 3. One of the following is NOT how you give chest compression to a newborn baby | A. Be sure chest is inflating with breaths  B. Use one hand C. Thumbs on sternum between nipples D. Count 3 compressions per breath from Ambo bag | B |
|  | 4. When should you give chest compressions to a newborn baby? | A. If you've not managed to open the airway B. If no heartbeat at all C. If lungs are not inflated D. If heartrate is below 60bpm | D |
|  | 5. If the baby is stained with meconium, what should you do? | A. Open the airway immediately B. Remove meconium by suction C. Wash the baby D. Give inflation breaths right away | B |
| **How to care for a newborn** | 1. What will you need for a clean birth? | A. Peppermints B. Thermometer C. Senior medical qualifications D. Soap and water | D |
|  | 2. What do you NOT do as soon as the baby is born? | A. Wipe and dry baby clean B. Keep baby warm C. Weigh baby D. Give baby to mother | C |
|  | 3. What do you need to cut the cord hygienically? | A. Special ointment B. A kitchen knife C. A sharp stone D. Clean, new razor blade | D |
|  | 4. Why encourage a baby to breastfeed as soon as it is born? | A. Stops breast milk going on clothes B. first milk strengthens baby against illness C. Keeps mother busy so you can relax | B |
|  | 5. What should happen in the baby's first days? | A. Stump will grow longer B. Baby will start walking C. Baby passes wee D. Baby should start talking | C |
|  | 6. What is NOT a sign to tell you to take a newborn baby to the health centre? | A. If baby is small B. If baby has blue eyes or long hair C. If baby has a fit or seizure | B |
|  | 7. What is on your checklist to help a newborn and their mother? | A. Warm, dry baby B. Check baby breaths and moves while you dry C. Check football results for father D. Skin to skin with mother | C |
