# Supplementary material for: Acceptability and Potential Effectiveness of eHealth Tools for Training Primary Health Workers From Nigeria at Scale: Mixed Methods, Uncontrolled Before-and-After Study
Source: JMIR Mhealth Uhealth. 2021 Sep 16;9(9):e24182. doi: 10.2196/24182 (PMC8485189; doi:10.2196/24182)
Supplement: Multimedia Appendix 2 [file mhealth_v9i9e24182_app2.docx]

# **Multimedia Appendix 2: Interview guides**

**Evaluating impact of Extending Health Services to remote areas of Nigeria using SatCom**

**Midline Assessment**

**Generic Guide for Interviews for Heads of Health Facilities and Policy makers**

***Guidance notes***

**Before the interview**

- Specify how long IDIs will take. You should familiarize yourself with the question guide and mark priority questions for the interview on the interview guide accordingly.
- You should also be familiar with the definitions and the terminology used in the project glossary (if one exists). You should be ready to clarify certain concepts and what they mean to the respondents during the interview
- As the interviews will be audio-recorded, you should check that the recorder is working properly before going into the interview.
- Check that you have notebooks and pens for note-taking and/or laptop computers as appropriate.
- Take extra copies participant information sheet, informed consent and other key documents that are deemed useful for each interview.

**At the beginning of an interview**

- Introduce yourself and other researchers present (if applicable) to the respondent.
- Briefly explain the objectives of the research and obtain informed consent.
- Check the overall time available for the interview.
- Explain that this will be an open interview and ascertain that respondent understand what is expected of them e.g. “In your own words, we would like you to tell us your personal knowledge and experiences of providing oversight within the health systems in Nigeria.”

**During the interview**

- Although we are interested in the respondent’s own views and experiences, for the purpose of the interviews, you are allowed to tactfully interrupt respondents to clarify issues related to the initial working theories or provide prompts. But do not try to always fill the silence with a next question or probe—your respondent may need some time to think about the issue.
- The bullet points in the next page provide a checklist of possible issues to raise. Use your judgement and ask these bullet points only if the conversation is faltering, or if the respondent is deviating too much from the question you asked them. Please note that some bullet points may not be applicable to some respondents.
- Keep track of the questions asked;
- Take note of non-verbal clues that might be important for analysis.
- If the interview is not being recorded (due to respondent refusal, or equipment failure), the note-taker's priority is to summarise the content of the interview
- During the interview, the interviewer should listen and be prepared to ask follow-up questions that further clarify issues.
- Use probes carefully; you do not need to ask all probes for all questions. Use your judgement and apply probes when the respondent is not answering fully or freely, or if the respondent doesn’t appear to understand the question.
- Probe for specific examples from respondent’s experience in support of her/his statements where possible.
- If respondent is not answering a question in detail, ask ‘give-me-an-example’ type question to complement the question route.
- Always let the respondent finish a phrase; try not to interrupt her/him
- Use the interview guide flexibly, allowing the respondent to address the questions in any order but ensuring that the key issues are covered.

**After the interview**

- Thank the respondent/interviewee for their participation
- Remember to catalogue the tape/file (if you are recording)
- During debriefing, reflect on, discuss in the research team and clearly note down any other issues (e.g. respondent’s mood during the pauses, your general impressions of the interview, good questions to ask) that may be important for future interviews, and the data analysis.
- Transcription if possible should start the day of the interview or at most the next day.

| **Information area** | **Example questions and probes** | ***Comments*** |
| --- | --- | --- |
| **Introduction by respondent** | | |
|  | For the tape, can you introduce yourself, and describe your work/what you do? | This question is to get respondents talking |
| **Section A:**  **Staff training** | 1. Before the use of video training in this facility (or LGA), how were your staff trained? | Historical insight into training practice in LGA or health facility |
|  | 1. Before the use of video training in this facility (or LGA), what was the regularity of staff training (monthly, yearly, 2-yearly etc.) and why? please give reasons for your answer   **Probe:** Categories of staff that benefited from regular training and why?   - Categories of staff that were left out and why? - How trainings were conducted (logistics, for example - Where the trainings were conducted? - Who or which organization provided the trainings and why? |  |
|  | 1. In your opinion, how has using video training for staff training in this facility (or LGA) compared with using face-to-face training, meetings, workshops, seminars etc.?   **Probe:** How did the adoption of video training affect:   - The duration or timing of training programmes - Availability of staff at their duty posts during training - Financial reward for attending trainings, workshops and seminar. | Comparison of e-Health application to other modes of training |
|  | 1. What difficulties or challenges did **you, anyone** or **your facility** face regarding using video training to train health workers in this facility (or LGA)?   **Probes:**   - Did challenge(s) mentioned above affect standard of healthcare delivery in your facility? If yes, how? - How was the challenge addressed or overcome? | Challenges/ difficulties experienced with using VTR to train staff |
|  | 1. In your opinion, how did the use of video training affect how health staff are trained in this facility (or LGA)? please give reasons for your answers   ***Probe:*** Both positive and negative response are Ok here. | Effect of e-Health application on staff training |
| **Section B:**  **Data management** | 1. In your opinion, how has the use of CliniPAK application affected how data is collected and managed in this facility (or LGA).   **Probe:** Both positive and negative responses are Ok here. | Effect of e-Health App on data collection and managed |
|  | 1. How has the use of CliniPAK application affected who/which department/unit is responsible for collecting and/or analyzing the data in this facility?   **Probe:** Has this changed? If yes, how? | Effect of e-Health App on responsibility for collecting and analyzing data |
|  | 1. How has the use of CliniPAK application affected the way health services data is transmitted and reported to the LGA headquarters?   **Probe:** Has this changed? If yes, how? | Effect of e-Health App on data transmission and reporting |
|  | 1. How has the introduction of CliniPAK application affected the way health service data is validated by the LGA headquarters?   **Probe:** Has the way of validating data changed? If yes, how? | Effect of e-Health App on data validation |
|  | 1. How has the introduction of CliniPAK Application affected who/ which department/unit was responsible for transmitting and/or reporting the data in this facility?   **Probe:** Has this changed? If yes, how? | Effect of e-Health App responsibility for data transmission and reporting |
| **Section C:**  **Supervision** | 1. In this facility, are FHWs supervised? If yes, how are they currently supervised? Who supervises them? And why are they supervised?   **Probe:** The frequency of supervision (daily, weekly, monthly, yearly etc.) and why? please give reasons for your answer | Insight into practice of supervision |
|  | 1. What difficulties or challenges did you face regarding supervision of health workers?   **Probes:**   - Did challenge(s) mentioned above affect standard of healthcare delivery in your facility? If yes, how? - How was the challenge addressed or overcome? |  |
|  | 1. What recommendations do you give to improve how supervision is done in this health facility? |  |
| **Section D:**  **Decision-making** | 1. Since the introduction of CliniPAK in this facility/LGA, have you used eHealth data available from CliniPAK for decision-making in your health facility/LGA?   **Probe:**   - Details about decisions that were informed by CliniPAK data - Has the implementation of CliniPAK influenced decision to adopt eHealth tools in this LGA/State? |  |
| **Section E:**  **Existing policies and programmes** | 1. From your experience, do you know of any policy or other projects in the LGA that promote or use e-health solutions for training FLHWs and or manage data?   **Probe:**   - Details about these policies or projects. - Year the policy/projects started - How the policy/projects is being implemented - The performance of such policy or projects |  |
|  | 1. Since the introduction of the VTR and CliniPAK in this LGA/state, has there been any policy document introduced or policy change in terms of using e-health solutions for health care delivery? If yes, give details.   **Probe:**   - When the policy document or policy change was introduced? - What informed such policy document/change? |  |
|  | 1. Since the introduction of the VTR project in this state, has there been expansion of use of VTR or CliniPAK to other health facilities (or LGAs) that you are aware of? If yes, give details.   **Probe:**   - When did the expansion to other facilities or LGA take place? - What informed such expansion of use of VTR and/or CliniPAK? - Do you know who/what organization funded the expansion? |  |
| **For Policymakers Only** | **General questions related to IPP Nigeria evaluation questions**  **Relevance of project:**   - How has the project affected the standard of healthcare in the State /LGA? - How are the project objectives related to State ICT and MNCH priorities?   **Effectiveness of project:**   - How has the project affected the health of women and their infants in this State/LGAs and communities?   **Efficiency of project:**   - How has the project affected government’s allocation of resources to FHWs training and data management?   **Impact of project:**   - What practical difference is the project making to health and healthcare in affected LGAs or communities? - What are positive and negative impacts of the project?   What are the primary and secondary long-term effects by the project, directly or indirectly, intended or unintended?  **Sustainability**   - How easy will it be for the LGAs and the state to take over project when donor input finishes e.g. from Inmarsat (SatCom)? |  |

**Evaluating impact of Extending Health Services to remote areas of Nigeria using SatCom**

**Midline Assessment**

**In-Depth Interview Guide for Frontline Health Workers**

***Guidance notes***

**Before the interview**

- Specify how long IDIs will take. You should familiarize yourself with the question guide and mark priority questions for the interview on the interview guide accordingly.
- You should also be familiar with the definitions and the terminology used in the project glossary (if one exists). You should be ready to clarify certain concepts and what they mean to the respondents during the interview
- As the interviews will be audio-recorded, you should check that the recorder is working properly before going into the interview.
- Check that you have notebooks and pens for note-taking and/or laptop computers as appropriate.
- Take extra copies participant information sheet, informed consent and other key documents that are deemed useful for each interview.

**At the beginning of an interview**

- Introduce yourself and other researchers present (if applicable) to the respondent.
- Briefly explain the objectives of the research and obtain informed consent.
- Check the overall time available for the interview.
- Explain that this will be an open interview and ascertain that respondent understand what is expected of them e.g. “In your own words, we would like you to tell us your personal knowledge and experiences of working within the health systems in Nigeria.”

**During the interview**

- Although we are interested in the respondent’s own views and experiences, for the purpose of the interviews, you are allowed to tactfully interrupt respondents to clarify issues related to the initial working theories or provide prompts. But do not try to always fill the silence with a next question or probe—your respondent may need some time to think about the issue.
- The bullet points in the next page provide a checklist of possible issues to raise. Use your judgement and ask these bullet points only if the conversation is faltering, or if the respondent is deviating too much from the question you asked them. Please note that some bullet points may not be applicable to some respondents.
- Keep track of the questions asked;
- Take note of non-verbal clues that might be important for analysis.
- If the interview is not being recorded (due to respondent refusal, or equipment failure), the note-taker's priority is to summarise the content of the interview
- During the interview, the interviewer should listen and be prepared to ask follow-up questions that further clarify issues.
- Use probes carefully; you do not need to ask all probes for all questions. Use your judgement and apply probes when the respondent is not answering fully or freely, or if the respondent doesn’t appear to understand the question.
- Probe for specific examples from respondent’s experience in support of her/his statements where possible.
- If the respondent is not answering a question in detail, ask ‘give-me-an-example’ type question to complement the question route.
- Always let the respondent finish a phrase; try not to interrupt her/him
- Use the interview guide flexibly, allowing the respondent to address the questions in any order but ensuring that the key issues are covered.

**After the interview**

- Thank the respondent/interviewee for their participation
- Remember to catalogue the tape/file (if you are recording)
- During debriefing, reflect on, discuss in the research team and clearly note down any other issues (e.g. respondent’s mood during the pauses, your general impressions of the interview, good questions to ask) that may be important for future interviews, and the data analysis.
- Transcription if possible should start the day of the interview or at most the next day.

| **Information area** | **Example questions and probes** | ***Comments*** |
| --- | --- | --- |
| **Introduction by respondent** | | |
|  | For the tape, can you introduce yourself, and describe your work/what you do in this facility (CHEW, mid-wife, nurse, doctor etc.)?  **Probe:** Length of time staff has worked in this facility (in years) | This question is to get respondents talking |
| **Section A:**  **Staff training** | 1. Before the introduction of video training in this facility how were staff trained? | Historical insight into training practice in health facility |
|  | 1. Before the introduction of video training in this facility, what was the regularity of staff training (monthly, yearly, 2-yearly etc.) and why? please give reasons for your answer   **Probe:** Categories of staff that benefited from regular training and why?   - Categories of staff that were left out and why? - How trainings were conducted (logistics, for example) - Where the trainings were conducted? - Who or which organization provided the trainings and why? |  |
|  | 1. In your opinion, how has using video training to train staff in this facility compared with using face-to-face training, workshops, seminars etc.?   **Probe:** How did the adoption of video training affect:   - The duration or timing of training programmes - Availability of staff at their duty posts during training - Financial reward for attending trainings, workshops and seminar. | Comparison of e-Health application to other modes of training |
|  | 1. What difficulties or challenges did **you, anyone** or **your facility** face regarding using video training to train health workers in this facility (or LGA)?   **Probes:**   - Did challenge(s) mentioned above affect standard of healthcare delivery in your facility? If yes, how? - How was the challenge addressed or overcome? | Challenges/ difficulties experienced with using VTR to train staff |
|  | 1. In your opinion, how did the use of video training affect how health staff are trained in this facility (or LGA)? please give reasons for your answers   ***Probe:*** Both positive and negative response are Ok here. | Effect of e-Health application on staff training |
| **Section B:**  **Benefits of VTR and CliniPAK to frontline health workers** | 1. From your experience, has participating in VTR and/or using CliniPAK been beneficial to you? If yes, what benefits you refer to.   ***Probe:*** *Benefits of VTR can include but not limited to*   - Improved understanding of MNCH subject matter - Preparing FHWs for their role in healthcare delivery - Exposing FHWs to high quality training content - Increased knowledge and skills - Helping FHWs to perform their tasks - Improvements in health of women and their infants in this LGA - Improvements in standard of healthcare provided in this facility? - Other benefits? | Views of benefits of participation in VTR and using CliniPAK |
|  | 1. How has your exposure to VTR training and/or exposure to using CliniPAK affected your confidence and self-esteem in discharging their duties? Please explain why this is the case.   ***Probe:***   - How has participating in VTR training affected your self-confidence? Please explain. - How has participating in the VTR affected your motivation to work as a health worker? - In your opinion, why has VTR affected you in this way? Please explain. | Effect of eHealth tools on self-confidence, self-esteem and staff motivation to work |
|  | 1. How has your exposure to VTR training and/or using CliniPAK affected your job satisfaction? Please explain why this is the case.   ***Probe:***   - How has participating in VTR training and/or using CliniPAK affected job satisfaction? Please explain. - Why has VTR and/or CliniPAK had this effect? Please explain. | Effect of eHealth tools on job satisfaction |
|  | 1. What can be done to improve use of VTR for health worker training? | Recommendations for improving VTR use |
| **Section C:**  **Data management** | 1. In your opinion, how has the use of CliniPAK application affected how data is collected and managed in this facility?   **Probe:** Both positive and negative responses are Ok here. | Effect of e-Health App on data collection and managed |
|  | 1. How has the use of CliniPAK application affected who/which unit is responsible for collecting and/or analyzing the data in this facility?   **Probe:** Has this changed? If yes, how? | Effect of e-Health App on responsibility for collecting and analyzing data |
|  | 1. How has the use of CliniPAK application affected the way health services data is transmitted and reported to the LGA headquarters?   **Probe:** Has this changed? If yes, how? | Effect of e-Health App on data transmission and reporting |
|  | 1. How has the introduction of CliniPAK Application affected who/ which department/unit was responsible for transmitting and/or reporting the data in this facility?   **Probe:** Has this changed? If yes, how? | Effect of e-Health App responsibility for data transmission and reporting |
|  | 1. How has the use of CliniPAK application affected the way health service data is validated by the LGA headquarters?   **Probe:** Has the way of validating data changed? If yes, how? | Effect of e-Health App on data validation |
| **Section D:**  **Supervision** | 1. Regarding staff supervision, have you been supervised in this health facility in the past 1 year? If yes, how often were you supervised? Who supervises you? And why do you think you are being supervised?   **Probe:**   - Frequency of supervision (daily, weekly, monthly, yearly etc.) and why? please give reasons for your answer - What aspects of your work were supervised? - Did supervision involve skills check? If yes, what skills were checked? | Experience of being supervised |
|  | 1. Do you derive benefits from being supervised? If yes, what benefits?   **Probes:**   - What benefits do you derive from being supervised? | Benefits of supervision to FHW |
|  | 1. What recommendations do you give to improve how supervision is done in this health facility? |  |
